# P-1208. In Vitro Activity of Sulopenem and Comparator Agents Against U.S. Enterobacterales Clinical Isolates, SENTRY Antimicrobial Surveillance Program, 2023

**DOI:** 10.1093/ofid/ofaf695.1401

**Published:** 2026-01-11

**Authors:** Steven I Aronin, Michael D Huband, Holly Becker, Kelley A Fedler, Michael Dunne

**Affiliations:** Iterum Therapeutics, Old Saybrook, CT; Element, North Liberty, IA; Element Materials Technology/Jones Microbiology Institute, NORTH LIBERTY, Iowa; Element, North Liberty, IA

## Abstract

**Background:**

Sulopenem is a thiopenem antibacterial with an oral and parenteral formulation. Sulopenem etzadroxil/probenecid, the oral formulation, was recently approved by the US FDA for the treatment of women with uncomplicated urinary tract infection. This study evaluated the in vitro activity of sulopenem and comparator agents against contemporary Enterobacterales clinical isolates predominantly from patients with urinary tract infections.

**Figure:**
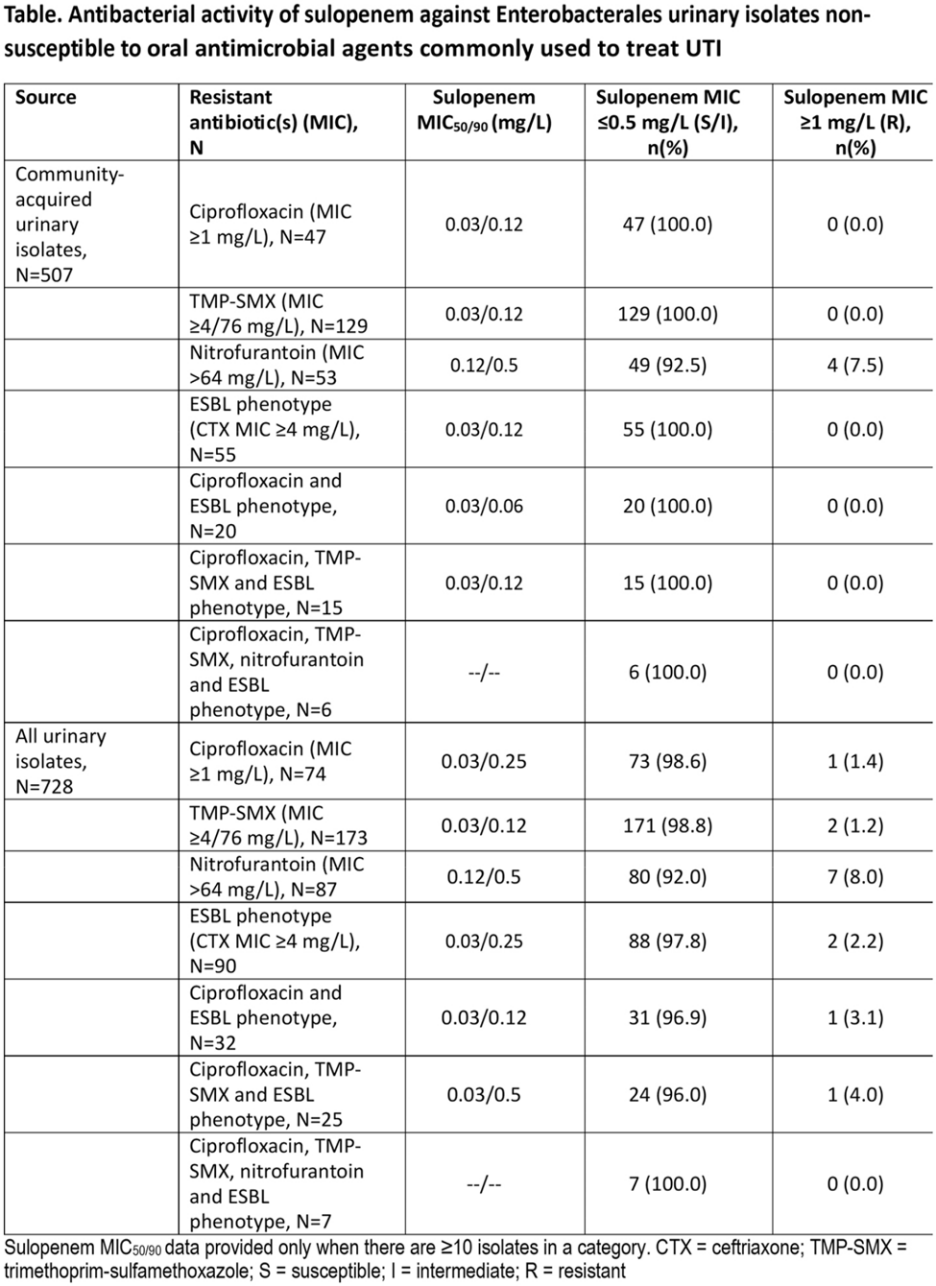

**Methods:**

A contemporary collection of 1,086 community- and nosocomial-acquired Enterobacterales isolates was assembled from US medical centers. Isolates were susceptibility tested using the CLSI broth microdilution reference method.

**Results:**

Sulopenem demonstrated potent in vitro antimicrobial activity (MIC_50/90_, 0.03/0.25 mg/L) against Enterobacterales isolates regardless of infection type, inhibiting 98.0% of isolates at ≤0.5 mg/L. This activity was conserved against resistant phenotypes including ESBL-phenotype *Escherichia coli* (MIC_50/90_, 0.03/0.06 mg/L) and ESBL-phenotype *Klebsiella pneumoniae* (MIC_50/90_, 0.06/0.12 mg/L). Sulopenem maintained activity against ciprofloxacin-, nitrofurantoin- and trimethoprim/sulfamethoxazole-non-susceptible subsets, including urinary isolates from patients in the community (MIC_50/90,_ 0.03-0.12/0.12-0.5 mg/L). Sulopenem also maintained activity against community-acquired ESBL-producing Enterobacterales urinary isolates non-susceptible to two or more oral antimicrobial agents commonly used to treat urinary tract infections.

**Conclusion:**

The potent in vitro activity of sulopenem against this large collection of contemporary Enterobacterales clinical isolates from multiple infection types supports its use in the treatment of uncomplicated urinary tract infection, as well as its further clinical evaluation in the treatment of other common bacterial infections demonstrating resistant phenotypes.

**Disclosures:**

Steven I. Aronin, MD, Iterum Therapeutics: Employee|Iterum Therapeutics: Stocks/Bonds (Public Company) Michael D. Huband, BS, Melinta Therapeutics: Advisor/Consultant|Melinta Therapeutics: Grant/Research Support Kelley A. Fedler, BS, Melinta Therapeutics: Grant/Research Support Michael Dunne, MD, Iterum Therapeutics: Board Member|Iterum Therapeutics: Employee|Iterum Therapeutics: Stocks/Bonds (Public Company)

